# Immobilization of Urease Nanoflowers on a Conjugated Polymer Thin Film for Enhanced Catalytic and Optoelectronic Performance

**DOI:** 10.1021/acsomega.5c10753

**Published:** 2026-05-24

**Authors:** Cléber Gomes de Jesus, Luciano Caseli, Laura Oliveira Péres

**Affiliations:** Laboratory of Hybrid Materials, Federal University of São Paulo, Diadema 09913-030, SP, Brazil

## Abstract

Enzyme–hybrid nanoflowers (EHN) have attracted substantial interest in biocatalysis due to their high surface area and enhanced catalytic activity compared with free enzymes. Although extensively studied in solution, their integration into solid supports remains limited. Here, thin films composed of urease/Cu^2+^ hybrid nanoflowers and the conjugated polymer (CP) poly­(9,9-dioctylfluorene-*co*-phenylene) were fabricated by drop-casting and spin-coating to evaluate their biocatalytic and optoelectronic potentials. The films were characterized by ultraviolet–visible, infrared, and fluorescence spectroscopy, as well as transmission electron microscopy. The immobilized nanoflowers retained ureolytic activity and, in some cases, outperformed polymer/bare-urease films. The Michaelis–Menten constant was comparable to values reported for enzyme–hybrid nanoflowers in solution, indicating a minimal loss of substrate affinity upon immobilization. Reusability tests revealed higher performance of nanoflower-based films over three cycles. These findings highlight the potential catalytic and optoelectronic properties of conjugated polymer/enzyme–hybrid nanoflower films, supporting their application in biosensors and functional devices.

## Graphical abstract

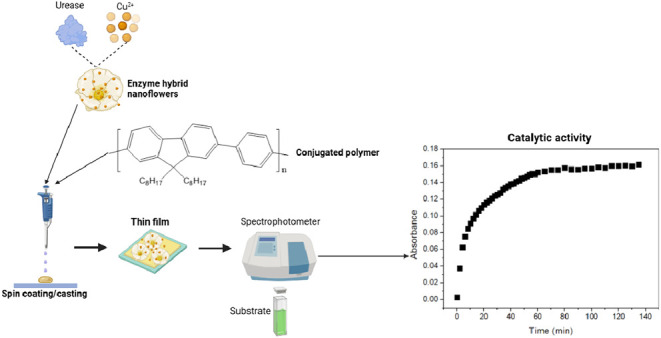

## 1 Introduction

Enzymes are central to modern biotechnology.^1–8^ Their incorporation into enzyme–hybrid nanoflowers (EHN),^9^ bioinorganic structures formed by combining enzymes with inorganic components, enhances surface area, stability, and functional performance^10–14^ while preserving enzymatic selectivity.^15^ For instance, Zhang et al. demonstrated a smartphone-based colorimetric biosensor using HRP-Cu_3_(PO_4_)_2_·3H_2_O nanoflowers for H_2_O_2_ detection.^16^

EHNs frequently exhibit higher catalytic activity than free enzymes due to optimized metal composition and morphology, which improve structural stabilization and substrate accessibility.^14,15,17^ However, when dispersed in solution, they are difficult to recover and reuse,^18^ limiting practical applications.

Immobilization in thin films offers a viable alternative, and for that, adsorption matrices like conjugated polymers (CPs) are an alternative. These compounds enhance stability, reduce leaching, and facilitate integration into devices.^6,18–27^ Enzymes can be immobilized by physical adsorption, cross-linking, covalent binding, or entrapment.^28–30^ Among these, physical adsorption is simple and scalable, although partial desorption may occur.

Fluorene- and phenylene-based CPs are particularly attractive due to their stability and high quantum yield.^31^ In our previous work,^6^ enzymatic films blended with poly­(fluorene-phenylene) CP exhibited superior kinetic performance among several functionalized polymers, indicating that intrinsic polymer properties can modulate catalytic activity.

Conjugated polymers can function as both immobilization matrices and transducers. In the present study, however, the polymer acts primarily as a structural support for enzyme–hybrid nanoflowers, while sensing is based on a colorimetric response rather than direct electronic coupling.^12,13^

Although research on EHNs is expanding, their incorporation into conjugated polymer thin films has remained largely unexplored. Here, we report the fabrication of urease/Cu^2+^ hybrid nanoflowers embedded in a conjugated polymer thin film, providing catalytic efficiency with a structural stability. To our knowledge, this is the first demonstration of EHN immobilization within a conjugated polymer thin film, advancing the understanding and development of solid-state biosensing and catalytic platforms.

## 2 Materials and Methods

### 2.1 Synthesis of PFPh Copolymer

The conjugated copolymer poly­(9,9-dioctylfluorene-*co*-phenylene) (PFPh) was previously synthesized via the Suzuki route,^32^ being characterized by nuclear magnetic resonance (NMR), gel permeation chromatography (GPC) (see the Supporting Information), and Fourier transform infrared spectroscopy (FTIR) using KBr as a background (Shimadzu IRPrestige-21 equipment, with 128 scans, 4 cm^–1^ resolution). Polymer solutions in chloroform were analyzed by UV–vis absorption spectroscopy using a Cary 60 spectrophotometer and by fluorescence spectroscopy (Fluorog FL3C-22) to evaluate their optoelectronic properties, with excitation performed at the maximum absorption wavelength. A 1 cm path quartz cuvette was used to analyze the solutions. The same characterizations were redone to ensure the polymer maintains its optoelectronic characteristics.

### 2.2 Synthesis of Urease/Cu^2+^ Hybrid Nanoflowers

The best conditions for HNF synthesis were based on the methodology proposed by Somturk et al.^11^ Urease from Jack beans (Sigma-Aldrich) was dissolved in phosphate buffer solution (PBS, 0.01 M, pH 7.4) at a 0.02 g·mL^–1^ concentration with additional ∼140 mM of NaCl. 45 mL of this solution was mixed with 300 μL of a CuSO_4_ (pentahydrate, LabSynth) solution at a 120 mM concentration, and the mixture was incubated for 3 days at 4 °C. During the reaction, Cu^2+^ and PO_4_^3–^ (from the PBS) ions interact, forming Cu_3_(PO_4_)_2_ by electrostatic interaction, followed by the interaction of the nanocrystals with the aminic backbone of the enzyme (nucleation step).^11^ After incubation, the nanoflowers were washed with distilled water and centrifuged at 4400 rpm for 30 min 3 times. The collected HNF as a blue precipitate was set to dry under vacuum at room temperature. A 1 mg mL^–1^ urease–HNF dispersion was characterized by UV–vis spectroscopy (Cary 60 spectrophotometer), and the collected blue precipitate was analyzed by FTIR spectroscopy using a Shimadzu IRPrestige-21 spectrometer (128 scans, 4 cm^–1^ resolution) with attenuated total reflectance (ATR). Dynamic light scattering (DLS) was used to obtain the particle size distribution, and the ζ-potential was estimated as −37.81 mV (Zetasizer Pro, Malvern Instruments—He–Ne 4 mV laser at 633 nm).

### 2.3 Preparation of Thin Films

PFPh was solubilized in chloroform (LabSynth) at a 0.5 mg·mL^–1^ concentration, and the urease HNF powder was dispersed in PBS (0.01 M, pH 7.4) at 1 mg·mL^–1^ concentration. For the spin-coated films, a 1 × 2 cm^2^-sized quartz plate was used to add 50 μL of each solution in the equipment (Microtube A1 V1.1.2). Films with 1–3 layers were formed to observe the influence of the amount of material on catalytic and optoelectronic properties. The best parameters of rotation speed and rotation time were 3000 rpm, 45 s for PFPh and 2000 rpm, 35 s for the HNF.^6^ Drop-cast films were also assembled by adding the same volume of the solutions to the quartz plate and leaving to dry at 60 °C. To estimate mass deposition and desorption, the Sauerbray equation was applied using the quartz crystal microbalance (SRS—QCM200)^33^ for the spin-coated films, while an analytical balance was used for the cast films. These materials were then deposited onto a 2.54 cm diameter circular chrome/gold quartz crystal (Stanford Research System O100RX1), with 0.4 cm^2^ of active area (area of overlap between the two circular pad electrodes).

The UV–vis characterizations were performed in the Cary 60 spectrophotometer. For fluorescence spectroscopy, a Fluorog FL3C-22 spectrophotometer was used. Such techniques were employed to understand the optoelectronic properties of thin films and possible interlayer interactions. FTIR analysis for the solids was performed in the Shimadzu IRPrestige-21 equipment (128 scans, 4 cm^–1^ resolution). For the transmission electron microscopy of the nanoflowers, JEOL JEM 2100 equipment was used (200 kV, 0.23 nm resolution), and JEOL LV 6600 equipment was used for SEM/EDS analysis.

### 2.4 Enzymatic Analysis

As in previous experiments,^6,31^ the thin film was immersed in a quartz cuvette containing 1.5 mL of urea solution (500 μM) and 1.5 mL of bromocresol purple (0.015 mM). The urea catalysis was monitored with increasing absorbance at 588 nm for 90 min inside the spectrophotometer. Since an indirect colorimetric method was applied, the enzyme activity was recorded at min^–1^ since absorbance is unitless.^6^ To evaluate the Michaelis–Menten constant, different concentrations of urea from 100 to 900 μM were used to apply the Lineweaver–Burk analysis.^6,34^ For temperature-dependent experiments, the same methodology was applied, varying the internal temperature of the spectrophotometer as desired. The Levenberg–Marquardt method was applied to adjust the obtained curves in the Michaelis–Menten model. All the experiments were repeated at least 3 times to ensure reproducibility.

## 3 Results and Discussion

### 3.1 Characterization of PFPh and Urease/Cu^2+^ Hybrid Nanoflowers

The UV–vis absorption spectra of the urease HNF dispersion and the PFPh solution, shown in Figure S1, are consistent with previous studies.^32,35,36^ The spectral profile and absorbance maxima of the PFPh solution closely match values reported in the literature.^6,37^ Native urease typically exhibits a maximum absorption near 278 nm; however, as reported by Yan-Qing and Hong-Mei, the coordination of Cu^2+^ ions with urease induces a blueshift in this peak. This interaction likely explains the broadened and shifted absorption band observed in the urease HNF spectrum.^38^

The FTIR analysis shown in Figure 1 reveals characteristic bands of PFPh that are consistent with previous studies. Noteworthy signals include those attributed to the fluorene group at 810 and 1460 cm^–1^ (C=C stretching), the aliphatic side chains at 2852 and 2926 cm^–1^ (C–H stretching for CH_2_ groups, symmetric and asymmetric, respectively), and the C–H stretching modes of the fluorene and phenylene rings at 3030 cm^–1^.^6,31,39^ For the urease HNF, the band at 1140 cm^–1^ corresponds to Cu–OH bending vibrations, and the discrete peak at 988 cm^–1^ is related to stretching vibrations of PO_4_,^3,40^ while the band at 1625 cm^–1^ may be attributed to structural water (O–H bending) or the C=O stretching from the Amide I band present in the urease protein,^40,41^ related to β-sheet structures. The broad absorption around 3400 cm^–1^ is also associated with structural water.^41^ In the spectrum of the 50:50 physical mixture of PFPh and urease HNF (green curve in Figure 1), bands corresponding to both components are clearly observed, suggesting physical interaction between the materials in the solid state.

**Figure 1:**
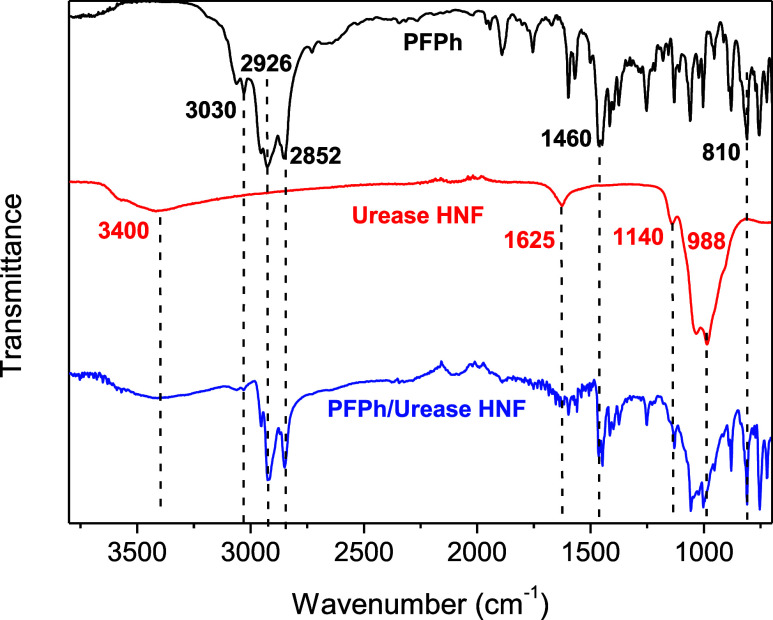
FTIR spectra of the PFPh, urease HNF, and PFPh/urease HNF powders. Transmittance values were vertically shifted for clarity.

Urease–Cu^2+^ hybrid nanoflowers are immobilized in the PFPh matrix mainly by physical adsorption, since no functionalization or cross-linking was applied. Retention likely arises from van der Waals and hydrophobic interactions, with possible minor CH−π or π–π contributions. As PFPh is neutral and nonfunctionalized, electrostatic interactions are negligible. FTIR data (Figure S4) show no new bands, confirming the absence of covalent bonding and preservation of both components’ chemical structures.

### 3.2 Film Characterization

Figure 2 shows thin film formation by spin-coating and casting. Spin-coating uses centrifugal force for uniform spreading and solvent evaporation, while casting involves solution spreading, followed by oven drying.

**Figure 2:**
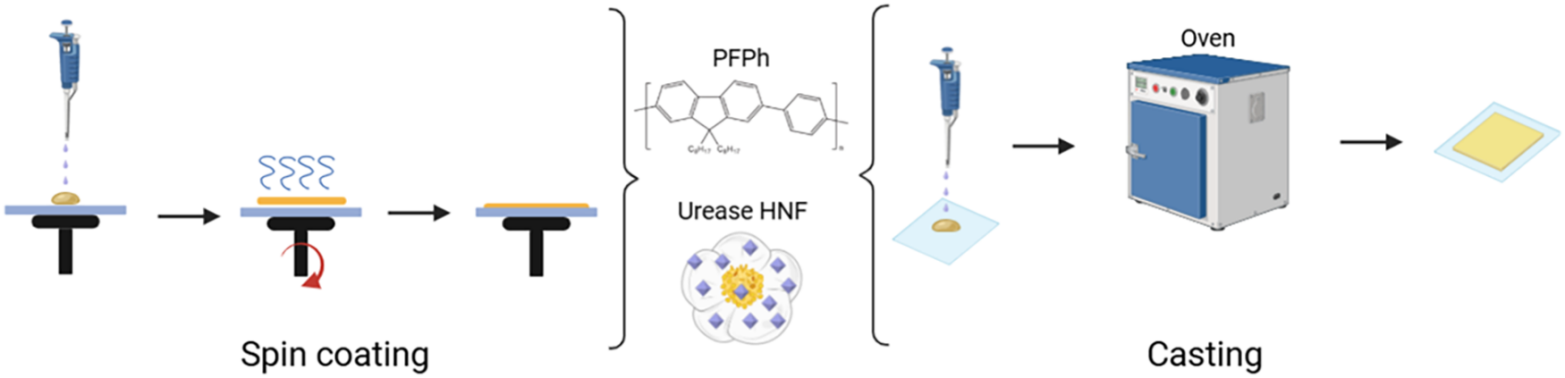
Scheme of thin films formation by spin-coating (left) and casting (right) methodologies.

For spin-coated films, Figure 3A shows that urease HNF alone exhibits negligible absorption, indicating poor retention on quartz and the need for a polymer matrix. In contrast, PFPh/urease HNF films display two absorption bands, confirming incorporation of both components and physical adsorption by PFPh.^6^ Absorbance increases with layer number, accompanied by a blueshift (HNF) and redshift (PFPh), likely due to conformational changes and aggregation in the solid state.^24,32^

**Figure 3:**
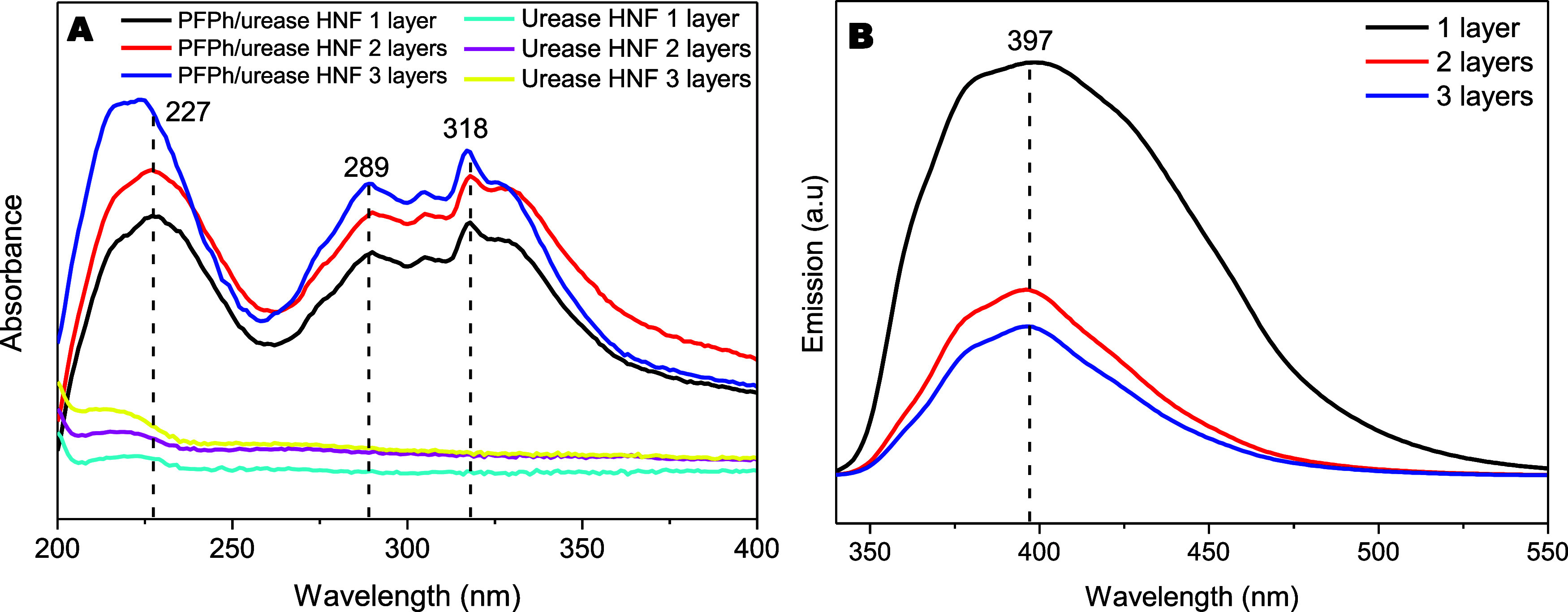
(A) UV–vis spectra for the PFPh/urease HNF and urease HNF spin-coated films with 1–3 layers and (B) emission spectra of the PFPh/urease HNF spin-coated films.

In the emission spectra of the films (Figure 3B), however, no significant shift in the peak position was observed. Instead, a decrease in the emission intensity occurred with increasing film layers. While the maximum emission wavelength remained consistent with previous reports,^6^ the progressive incorporation of urease HNF likely induced either the material leaching as additional layers were deposited or a quenching effect, suppressing PFPh fluorescence through nonradiative energy transfer or aggregation-induced quenching.^32,42^

Mass quantification of the spin-coated films was performed by using the QCM technique, as indicated by the surface density ratios presented in Table 1. For the first layer, the urease system exhibited a higher total surface density. However, the deposition of additional layers resulted in a decrease in the measured mass, supporting the hypothesis of the partial leaching of previously deposited materials. This interpretation is consistent with the fluorescence results discussed earlier, which also indicate material loss during the sequential deposition process, with the increase in absorbance attributed to possible aggregation of enzyme–PFPh chains during spin-coating, which enhances optical absorption. The effect was less pronounced for the HNF system than that for bare urease, suggesting stronger retention of the nanoflower structures within the film.

**Table 1:** Material Quantification for the Spin-Coated and Cast Thin Films

spin-coated films	
system	surface density (μg/cm^2^)
PFPh/HNF 1 layer	8.4452
PFPh/HNF 2 layers	6.5371
PFPh/HNF 3 layers	3.7279
PFPh/urease 1 layer	10.5477
PFPh/urease 2 layers	1.0247
PFPh/urease 3 layers	0.9187

cast films	
system	catalyst mass (mg)
PFPh/HNF	1.03
PFPh/urease	0.81

Such behavior may arise from partial desorption of the upper layers due to the centrifugal forces inherent to the spin-coating process.^43^ In addition, the increase in the absorption intensity observed in some samples may be related to changes in film morphology, local aggregation of PFPh chains, or variations in optical scattering caused by structural rearrangements during deposition.

In this case, it is not possible to determine the exact mass of the catalyst, since spin-coating films composed solely of the enzyme/HNF cannot be obtained. For the cast films, the transferred mass was determined directly by using an analytical balance. The mass of the catalyst was then roughly estimated by subtracting the mass measured for the cast films of the pure polymer. Although this approach has limited precision, it was used as an approximate parameter to estimate the amount of the enzyme present in the films.

TEM and SEM analyses revealed the morphology and aggregation of the nanoflowers. Early synthesis stages show petal-like structures (Figure 4A), which evolve into fully formed flower-shaped particles through petal interactions and folding (Figure 4B). SEM images (Figure 4C,D) highlight their porous internal structure. EDS mapping (Figure S3) confirms the presence of both organic and inorganic elements, verifying the successful incorporation. The nanoflower size and shape depend on the enzyme and metal ions used, as the crystal growth and protein structure influence the final morphology.^9,11,15,44^ The size distribution was according to previous reports using this synthesis, as shown in Figure S5.^11^

**Figure 4:**
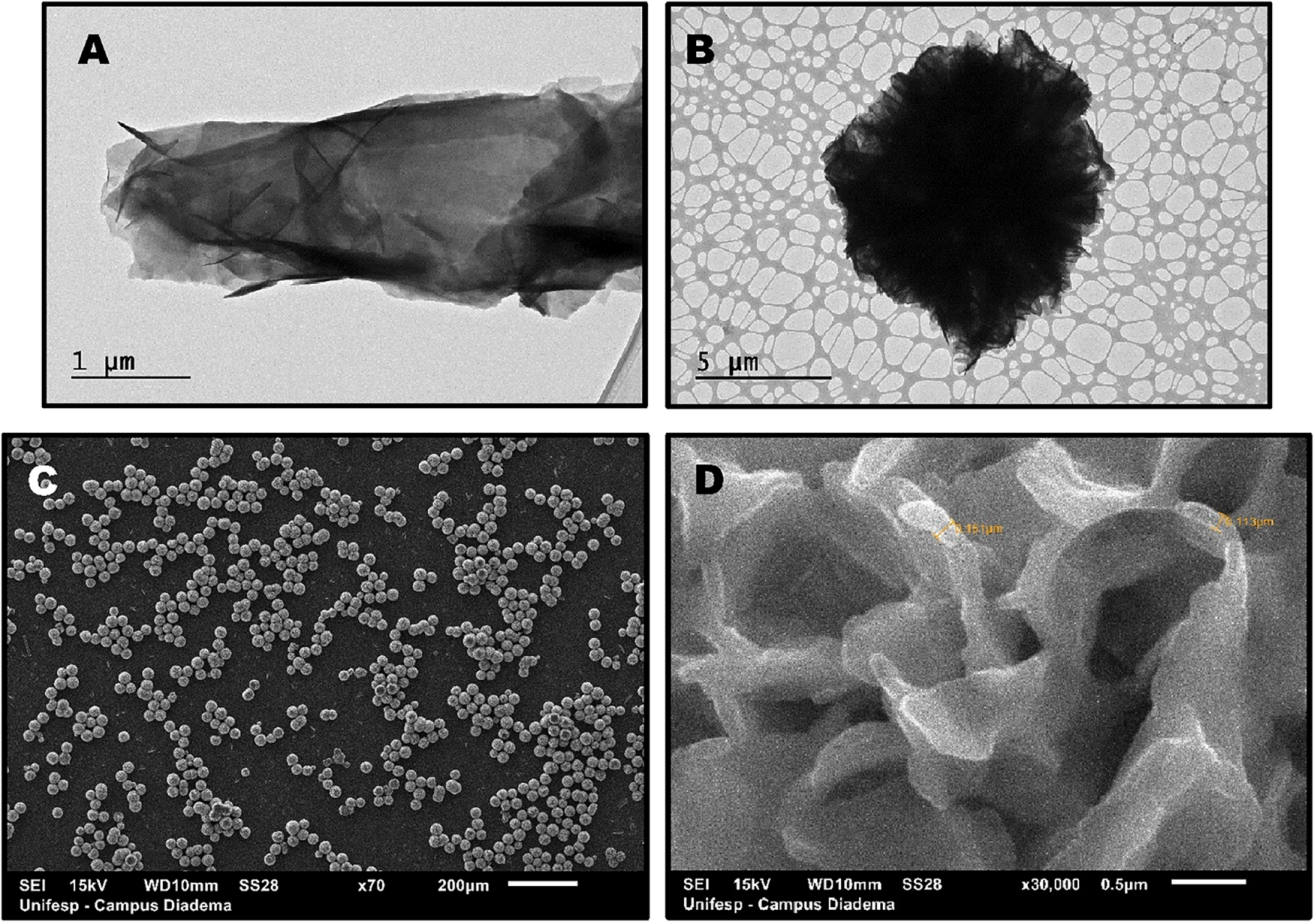
TEM images of the urease HNF with the amplification of (A) 1 μm, (B) 5 μm and SEM images of cast films containing immobilzed urease HNF with the amplification of (C) 200 μm and (D) 0.5 μm.

### 3.3 Enzymatic Analysis

As shown in Figure 5, urease catalyzes urea hydrolysis, generating ammonia and carbon dioxide.^45^ Starting at pH 6, ammonia increases the pH, causing bromocresol purple deprotonation and shifting its absorption from 430 to 588 nm.^6,31^ The reaction is monitored by the absorbance increase at 588 nm, following Michaelis–Menten kinetics.^6,46,47^

**Figure 5:**
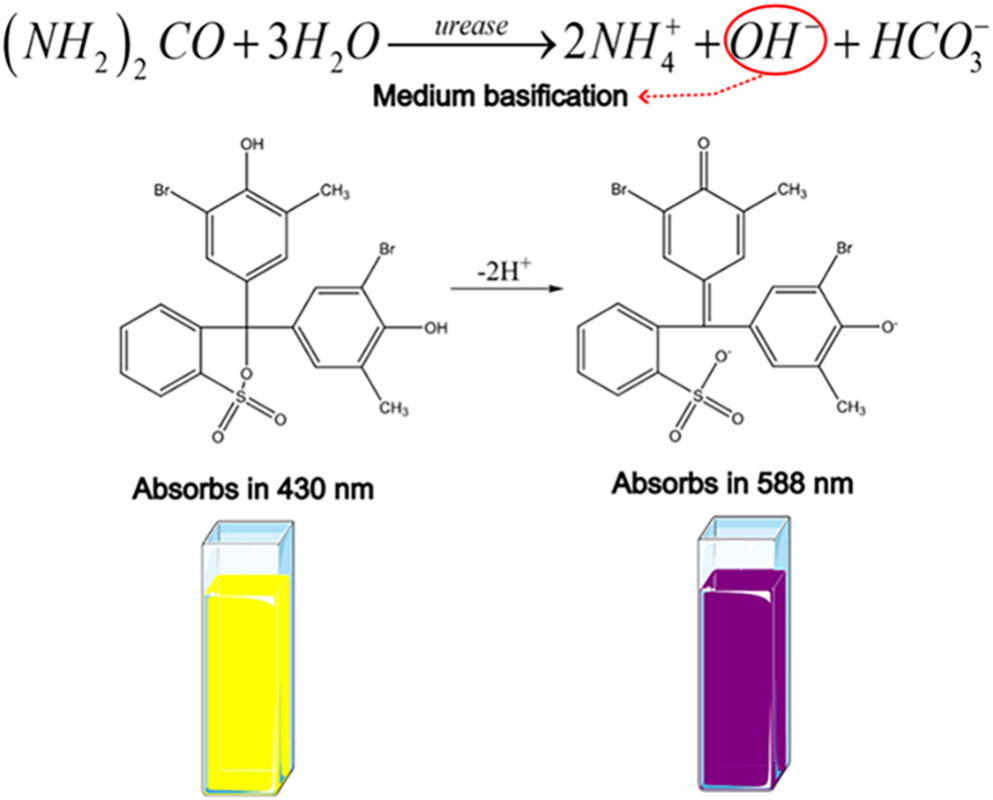
Strategy for urea hydrolysis monitoring using UV–vis spectroscopy.

To investigate the effect of miniaturization on enzymatic performance, activity assays were conducted using two systems: (i) urease hybrid nanoflower (HNF) immobilized within the PFPh matrix and (ii) urease incorporated into the same PFPh matrix. Maintaining the conjugated polymer in both systems ensured that any observed differences could be directly attributed to the nanoflower architecture rather than to matrix effects. For catalytic assays, cast films with one layer were used as the amount of urease HNF/urease was greater than the spin-coated films (Table 1).

As shown in Figure 6, both systems presented a curve that fits the Michaelian kinetic profile, indicating that the immobilization process did not affect the catalytic mechanism of urease. Importantly, this result indicates that the hierarchical organization of the enzyme into hybrid nanoflowers enhances the structural stability and interfacial accessibility without compromising the intrinsic enzymatic pathway. These findings reinforce that the miniaturized, biomimetic architecture is promising for evaluating the improvement of the performance through physicochemical optimization of the microenvironment rather than through alteration of the catalytic mechanism itself.

**Figure 6:**
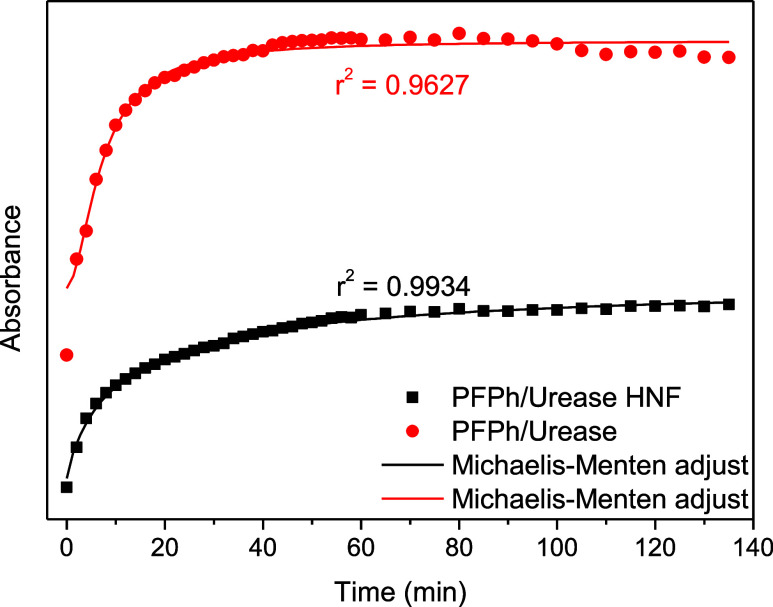
Michaelis–Menten fitting of the catalytic activity data for PFPh/urease HNF and PFPh/urease films during urea hydrolysis.

Enzymatic activity may decrease in spatially restricted or highly confined environments due to limited conformational flexibility and reduced mobility at the active site, which can impair substrate binding and turnover.^6,48^ For our case, urease is confined in two ways, bare or as a nanoflower. As shown in Table 2, the PFPh/HNF system exhibits values of relative activity comparable with that for bare urease, being higher at some specific substrate concentrations (500, 700, and 900 μM) compared to the PFPh/bare enzyme.

**Table 2:** Relative Enzyme Activity (Δ*A*_588_ s^–1^ mg^–1^ × 10^–3^) for the PFPh/urease and PFPh/urease HNF Films

urea concentration (μM)	PFPh/bare urease	PFPh/urease HNF
300	20.74	5.59
400	13.11	4.19
500	10.67	16.73
600	15.13	10.88
700	6.33	10.91
900	20.98	34.35

The enzymatic activity may increase in some cases for nanoflower structures due to their higher surface area and more open, petal-like morphology.^11,12,49^ Such structural features promote greater exposure of active sites and reduce diffusional barriers for urea transport within the film. In addition, the organized hybrid structure likely facilitates more efficient diffusion of the reaction product (ammonia) from the film to the surrounding solution, thereby minimizing local product accumulation and improving overall mass-transfer efficiency.^15,48,50^

Together, these factors indicate that the nanoflower-based immobilization strategy may mitigate the typical diffusion and conformational constraints associated with confined enzymatic systems, leading to an improved catalytic performance without altering the intrinsic reaction mechanism.

Although an increase in catalytic activity was observed at specific substrate concentrations, the PFPh/urease HNF film exhibited a higher apparent *K*_m_ (3.30 mM), as demonstrated in Figure S2, compared to the PFPh/urease film (0.32 mM). This shift likely arises from diffusional constraints and partial shielding of active sites within the nanoflower architecture and the polymer matrix, which can limit substrate accessibility and introduce mass-transfer effects.^31^

In contrast, the HNF-containing film displayed a markedly higher *V*_max_ (87.72 μM·min^–1^) than PFPh/urease (20.24 μM·min^–1^), demonstrating a substantially enhanced maximum catalytic rate.^34,51^ Because *K*_m_ represents the substrate concentration required to reach half of *V*_max_ and is commonly interpreted as an indicator of apparent enzyme–substrate affinity,^47,52^ the increase in *K*_m_ suggests a modest reduction in substrate affinity. However, the simultaneous and significant rise in *V*_max_ indicates that, once substrate diffusion barriers are overcome, the catalytic turnover is considerably improved. This behavior is consistent with an increased effective surface area and favorable enzyme orientation within the hybrid nanoflower structure, which can promote higher catalytic throughput.

For comparison, Somturk et al. reported a *K*_m_ value of 39 μM for free urease/Cu^2+^ nanoflowers,^11^ which is within the same order of magnitude considering differences in immobilization strategy and experimental conditions. Overall, immobilization within the PFPh matrix slightly increases the apparent *K*_m_, likely due to diffusional and interfacial effects, but preserves, and significantly enhances, the catalytic capacity relative to conventional PFPh/urease films, evidencing the synergistic effect between the conjugated polymer platform and the hybrid nanoflower architecture. The enhanced catalytic activity for the PFPh/urease HNF films at higher concentrations of urea may be related to the surface area of HNFs, providing a better access to active sites via allosteric effects and preventing saturation.^10,53^

Enzymes are temperature-sensitive with higher temperatures causing denaturation and loss of activity.^54^ In contrast, moderate heating increases molecular motion and catalytic rate.^6^ The temperature at which the activity is maximal is the optimum temperature; above it, the activity declines due to denaturation.^1^ In a previous study, we observed that the polymer matrix in the film led to a lower optimum temperature compared to free urease, probably due to changes in thermal propagation.^6^ In the present work, the PFPh/urease HNF system again exhibited an optimum temperature of 50 °C, suggesting that the film maintains robust activity within a temperature range suitable for applications involving environmental and biological samples, as shown in Figure 7.^55,56^

**Figure 7:**
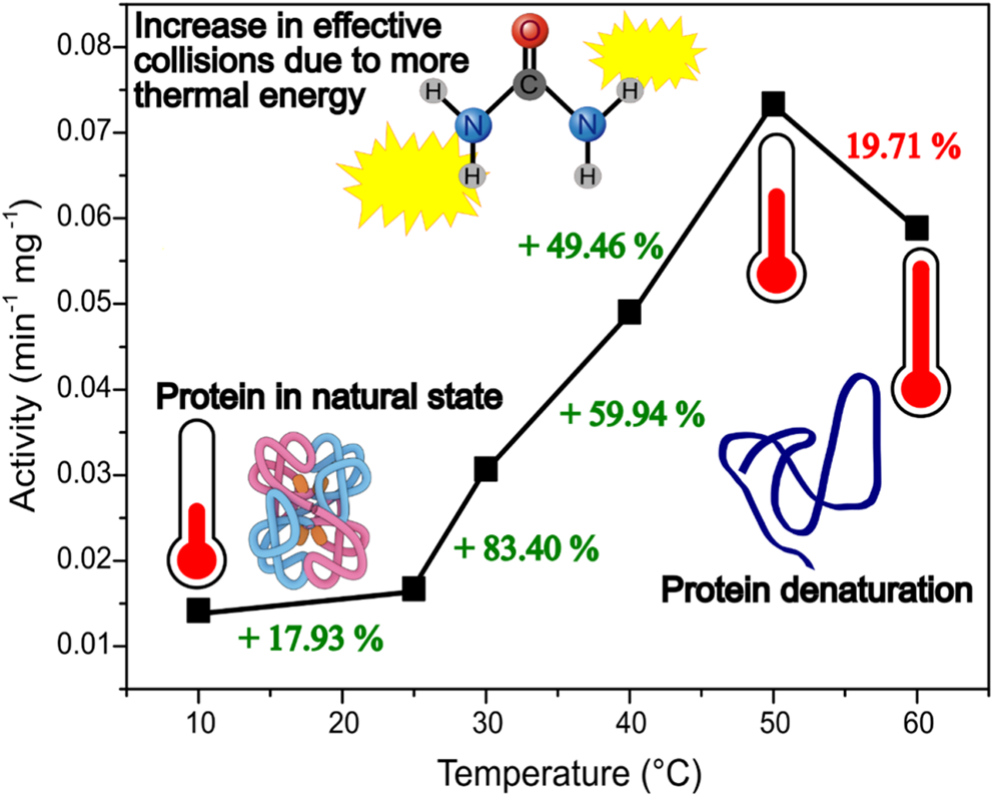
Variation of activity with temperature for the PFPh/urease HNF film.

The optimal temperature for immobilized urease in the PFPh/urease HNF film (50 °C) is slightly lower than that of free jack bean urease (55–65 °C).^57^ This shift likely results from the constrained microenvironment of the polymer matrix and nanoflower structure, which limit conformational flexibility and heat transfer. Such behavior is typical in immobilized systems and reflects structural and mass-transfer effects rather than changes in the intrinsic catalytic mechanism.

Although the urease nanoflower film showed enhanced catalytic and optoelectronic performance, device applications require long-term stability.^28–30,58^ Immobilization must therefore balance stability and activity, as strong binding can restrict enzyme mobility.^31^

Here, physical adsorption was used. Reusability tests (Figure 8) involved five consecutive 30 min cycles for urease HNF, PFPh/urease, and PFPh/urease HNF films. Between cycles, films were stored at 4 °C for 1 h to minimize thermal deactivation^54^ (see Figure 7). Activity measurements were performed without stirring.

**Figure 8:**
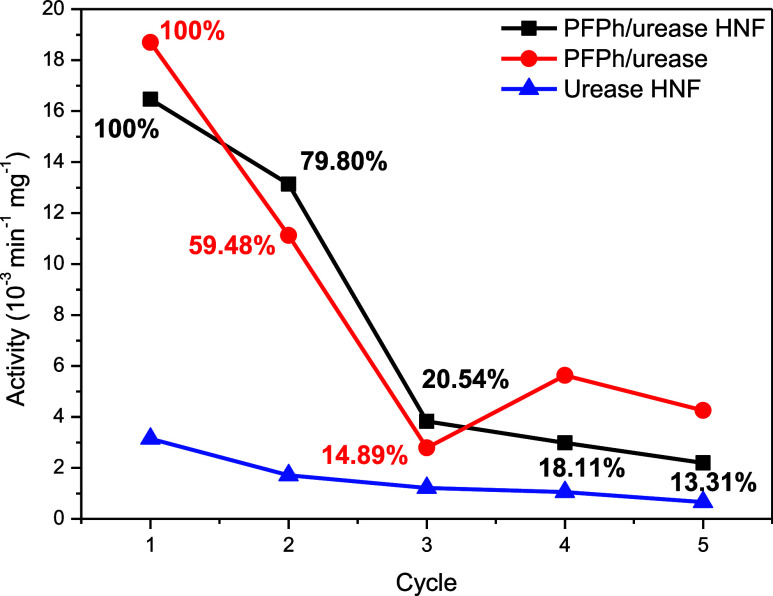
Reusability test in five cycles for urease HNF, PFPh/urease, and PFPh/urease HNF films.

The low activity of bare-urease HNF films confirms poor adhesion to quartz (Figure 3A), highlighting the need for a supporting matrix. Compared to PFPh/urease, the PFPh/urease HNF film shows higher activity up to the third cycle, suggesting partial entrapment of nanoflowers within the polymer matrix. The initial activity decline likely results from catalyst desorption, as amphiphilic enzymes can gradually diffuse into the aqueous medium.^59^ Leaching assays with spin-coated films indicated a mass loss around 1 μg (Figure S5), as expected for the weaker interactions between the components when using physical adsorption.^29^

The enhanced activity of PFPh films containing urease–Cu^2+^ nanoflowers is mainly attributed to the nanostructured architecture of EHNs. Since PFPh is hydrophobic, increased hydrophilicity is unlikely to contribute significantly. Instead, the hierarchical 3D structure improves the substrate mass transfer and enzyme distribution on the film surface. This is supported by Figure 8, where only the nanostructured system shows superior catalytic performance.

Although cross-linking density and molecular weight were not varied, PFPh’s physicochemical properties influence enzyme organization and mass transport. As a hydrophobic, rigid conjugated polymer, PFPh has limited swelling, restricting diffusion, and making the nanoflower architecture the main factor controlling catalytic performance. This aligns with our previous findings that polymer functional groups modulate enzyme immobilization and activity.^6^ Thus, even without varying these parameters, the PFPh structure contributes to the enzyme microenvironment.

## 4 Conclusion

This work introduces a novel strategy for fabricating functional thin films by immobilizing urease–Cu^2+^ hybrid nanoflowers (HNF) within a conjugated polymer matrix. To our knowledge, this is the first demonstration of EHN incorporation into a conjugated polymer thin film, moving beyond dispersion-based systems toward organized, solid-state platforms. The films were prepared by a simple physical adsorption method that preserved nanoflower morphology and activity. PFPh/urease HNF films showed enhanced catalytic performance at specific urea concentrations compared to PFPh/urease films, attributed to increased surface area and improved mass transfer. Thermal and reusability tests confirmed good stability over multiple cycles. Overall, this study establishes a new approach for integrating enzyme nanostructures into conjugated polymer thin films, providing a foundation for solid-state biosensors and catalytic devices.

## Supplementary Material



## Notes

Absorption spectra of the urease–HNF dispersion and PFPh solution (Figure S1); Lineweaver–Burk plots for the PFPh/urease–HNF and PFPh/urease systems (Figure S2); SEM images and EDS mapping of urease–HNF (Figure S3); FTIR spectra of PFPh/urease–HNF spin-coated films (Figure S4); desorption tests for HNF and urease films (Figure S5); QCM raw data for the spin-coated films (Table S1); and QCM raw data from the desorption tests (Table S2) (PDF)
