# Supplementary material for: Immobilization of Urease Nanoflowers on a Conjugated Polymer Thin Film for Enhanced Catalytic and Optoelectronic Performance
Source: ACS Omega. 2026 May 24;11(22):31949–56. doi: 10.1021/acsomega.5c10753 (PMC13261418; doi:10.1021/acsomega.5c10753)
Supplement: Supplementary file 1 [file ao5c10753_si_001.pdf]

Immobilization of Urease Nanoflowers on a Conjugated Polymer Thin Film for Enhanced Catalytic and Optoelectronic Performance

Cléber Gomes de Jesus, Luciano Caseli, Laura Oliveira Péres*

Laboratory of Hybrid Materials, Federal University of São Paulo, Diadema, SP, Brazil

Corresponding author: laura.peres@unifesp.br

Polymer synthesis

Poly[(9,9-dioctylfluorene)-co-phenylene] (PFPh) was synthesized using the Suzuki route [1], with 98.9% of yield. H^1 NMR (Ultrashield 300MHz, Bruker) ($CDCl_3$, ppm): 7.80–7.30 (m, 10H, Ar–H), 1.98–1.90 (m, 4H, CH_2); 1.05 (s, 24H, CH_2), 0.80–0.50 (m, 6H, CH_3) for PFPh. Gel Permeation Chromatography (GPC) using THF, polystyrene standard measurements showed: Mw = 1.7×10^3 g/mol and Mw/Mn = 1.1 for PFPh.

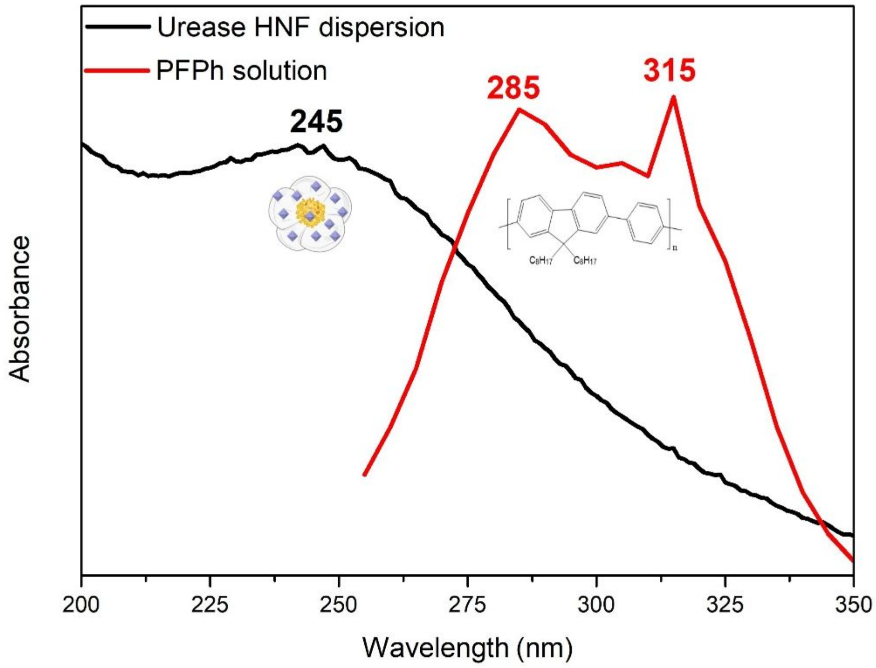

Figure S1. Absorption spectra for urease HNF dispersion (1 mg/mL) and PFPh solution (10^{-6} mg/mL).

Lineweaver-Burk plot

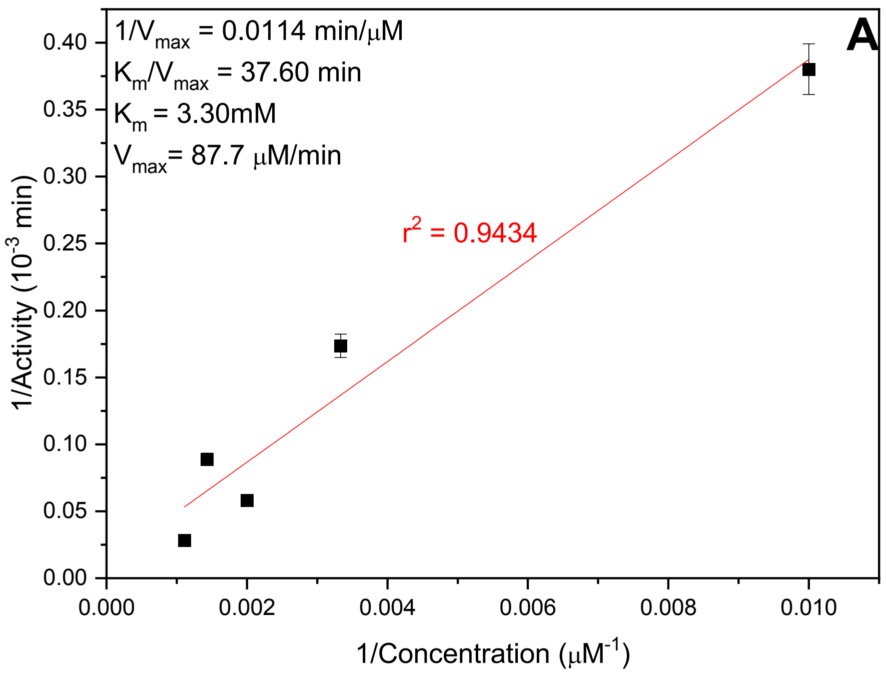

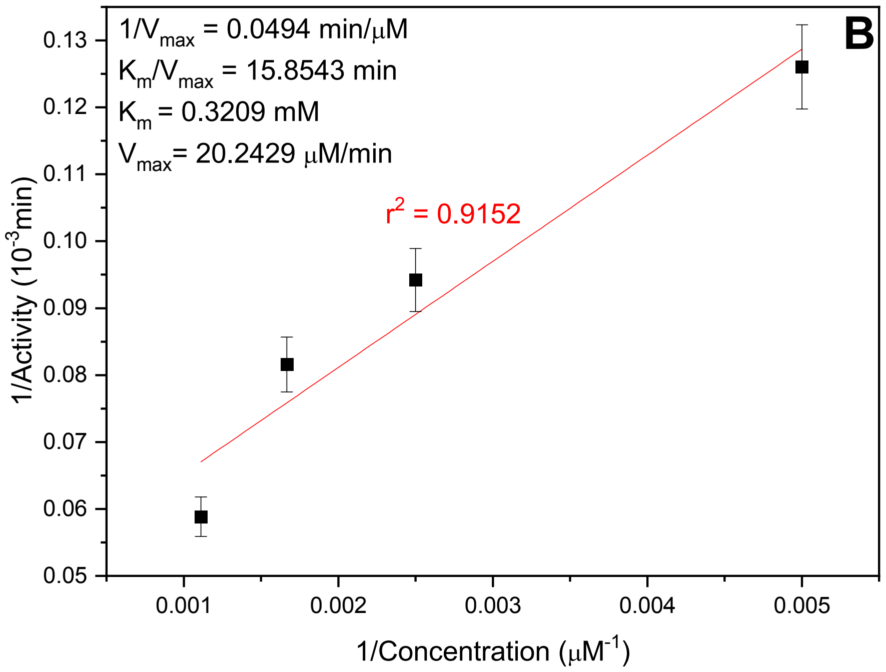

Figure S2. Lineweaver-Burk plot and Michaelis-Menten constant obtained for the (A) PFPh/urease HNF and (B) PFPh/urease systems during urea hydrolysis. The reliability of the measurements is within 5% as indicated by the error bars.

HNF and thin films characterization

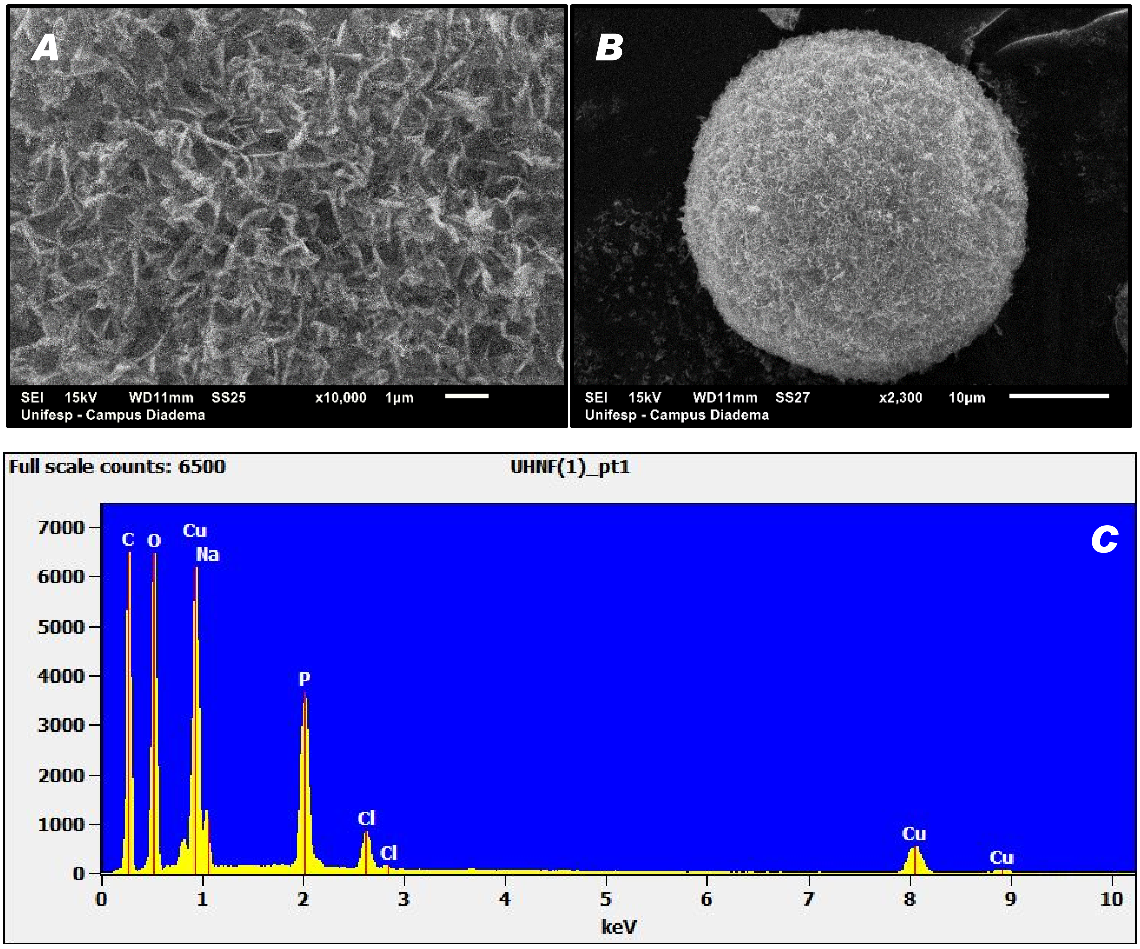

Figure S3. SEM images of non-immobilized urease HNF: amplification of (A) 1 µm and (b) 10 µm, and (c) EDS mapping of urease HNF.

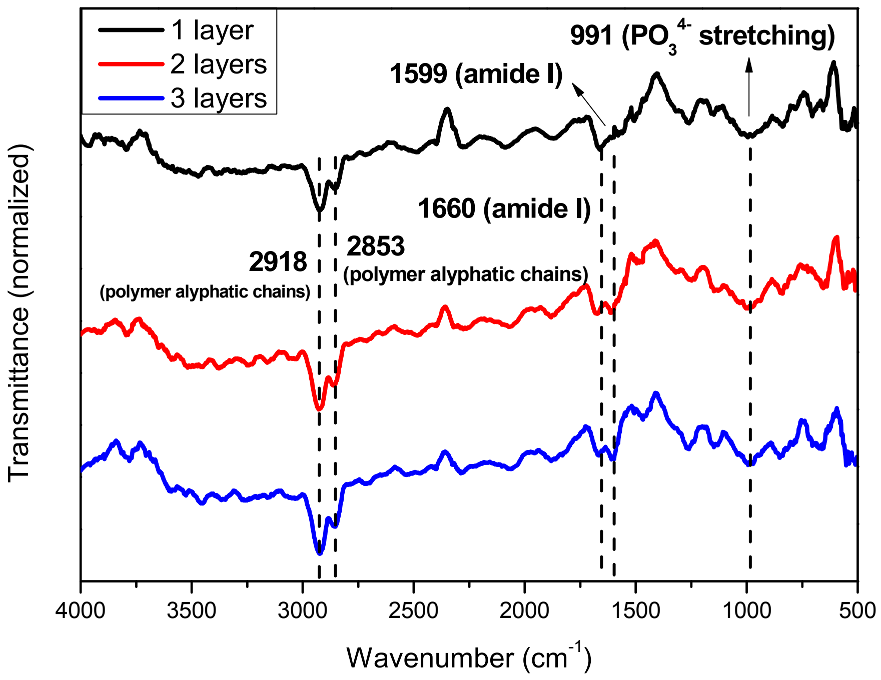

Figure S4. FTIR spectra for PFPh/urease HNF spin-coated films [2-6].

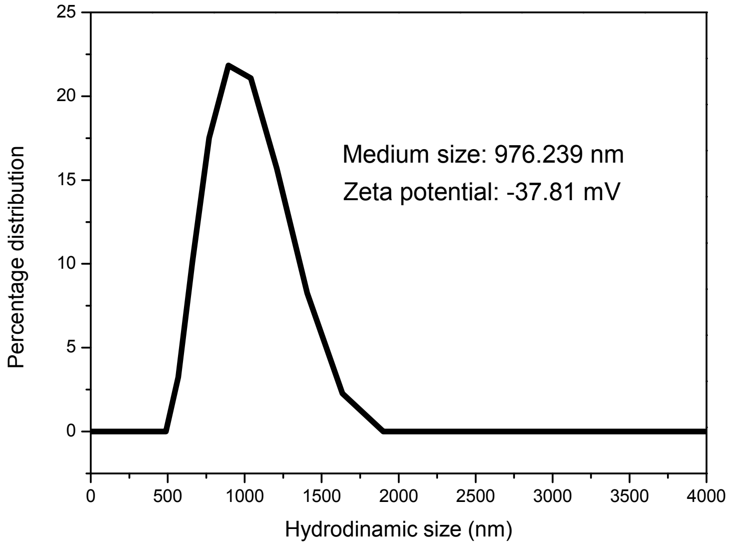

Figure S5. Size distribution and zeta potential of urease HNF.

QCM and desorption data

Table S1. QCM raw data for spin-coated films

Spin Coated Films	
System	**Frequency (Hz)**
Bare Substrate PFPh/HNF	5017079

PFPh/HNF 1 layer	5016601
PFPh/HNF 2 layers	5016231
PFPh/HNF 3 layers	5016020
Bare Substrate PFPh/bare enzyme	5017100
PFPh/urease 1 layer	5016503
PFPh/urease 2 layers	5016445
PFPh/urease 3 layers	5016393

Table S2. Desorption tests raw data (QCM).

Time (s)	HNF system – frequency (Hz)	Urease system – frequency (Hz)
0	5016001	5016165
10	5016010	5016169
20	5016052	5016163
30	5016070	5016161
40	5016087	5016158
50	5016101	5016158
60	5016109	5016158
70	5016114	5016159
80	5016119	5016158
90	5016125	5016160
100	5016131	5016161
110	5016136	5016161
120	5016141	5016160
130	5016147	5016160
140	5016149	5016160
150	5016150	5016160
160	5016151	5016160
170	5016153	5016160
180	5016153	5016159
190	5016156	5016159
200	5016158	5016158
210	5016160	5016158

220	5016161	5016158
230	5016163	5016158
240	5016163	5016157
250	5016164	5016156
260	5016164	5016155
270	5016163	5016155
280	5016163	5016155
290	5016163	5016153
300	5016163	5016153
310	5016163	5016153
320	5016162	5016152
330	5016164	5016152
340	5016164	5016151
350	5016163	5016151
360	5016164	5016151
370	5016166	5016150
380	5016170	5016149
390	5016170	5016149
400	5016172	5016149
410	5016173	5016148
420	5016173	5016147
430	5016173	5016146
440	5016173	5016144
450	5016173	5016144
460	5016173	5016143
470	5016174	5016142
480	5016173	5016142
490	5016173	5016141
500	5016174	5016141
510	5016173	5016140
520	5016174	5016139
530	5016176	5016139
540	5016174	5016138
550	5016174	5016137
560	5016174	5016136
570	5016174	5016134
580	5016175	5016133
590	5016175	5016133
600	5016174	5016131
630	5016174	5016126
660	5016174	5016120
690	5016174	5016116
720	5016175	5016113
750	5016177	5016113
780	5016178	5016110
810	5016178	5016108
840	5016178	5016103
870	5016178	5016099

900	5016179	5016094
930	5016178	5016092
960	5016177	5016091
990	5016178	5016089
1020	5016177	5016083
1050	5016176	5016076
1080	5016177	5016070
1110	5016176	5016069
1140	5016176	5016067
1170	5016176	5016066
1200	5016176	5016067
1230	5016175	5016066
1260	5016174	5016063
1290	5016174	5016059
1320	5016174	5016051
1350	5016172	5016047
1380	5016171	5016033
1410	5016171	5016017
1440	5016170	5016016
1470	5016170	5016014
1500	5016170	5016013
1530	5016168	5016010
1560	5016167	5016010
1590	5016166	5016011
1620	5016165	5016013
1650	5016163	5016014
1680	5016163	5016016
1710	5016163	5016018
1740	5016163	5016013
1770	5016163	5016010
1800	5016161	5016009
1830	5016161	5016009
1860	5016160	5016009
1890	5016159	5016010
1920	5016157	5016010
1950	5016157	5016011
1980	5016156	5016011
2010	5016154	5016013
2040	5016154	5016013
2070	5016151	5016012
2100	5016150	5016013
2130	5016147	5016012
2160	5016144	5016015
2190	5016144	5016018
2220	5016146	5016017
2250	5016146	5016017
2280	5016144	5016017
2310	5016143	5016019

2340	5016143	5016018
2370	5016141	5016019
2400	5016140	5016020
2430	5016139	5016020
2460	5016137	5016020
2490	5016136	5016020
2520	5016136	5016022
2550	5016136	5016023
2580	5016133	5016023
2610	5016131	5016023
2640	5016132	5016021
2670	5016131	5016019
2700	5016129	5016021
2730	5016127	5016024
2760	5016126	5016028
2790	5016126	5016029
2820	5016127	5016030
2850	5016127	5016033
2880	5016127	5016036
2910	5016124	5016039
2940	5016124	5016044
2970	5016123	5016047
3000	5016124	5016050
3030	5016123	5016053
3060	5016124	5016056
3090	5016124	5016059
3120	5016124	5016060
3150	5016124	5016064
3180	5016123	5016067
3210	5016121	5016069
3240	5016120	5016071
3270	5016120	5016074
3300	5016118	5016075
3330	5016118	5016076
3360	5016119	5016078
3390	5016120	5016079
3420	5016120	5016079
3450	5016120	5016080
3480	5016119	5016082
3510	5016119	5016084
3540	5016119	5016084
3570	5016118	5016084
3600	5016118	5016084

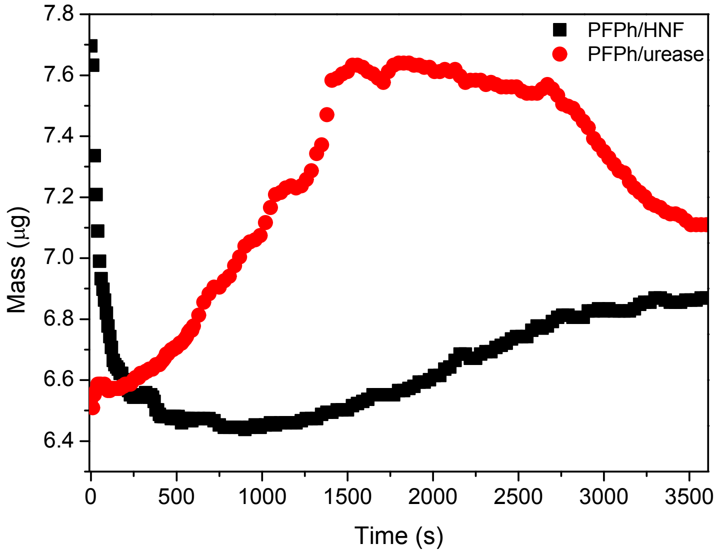

Figure S5. Desorption test for HNF and urease spin-coated films.

References:

[1] A. S. Menandro, J. C. Fernandes, H. P. M. Oliveira, and L. O. Péres, "Energy transfer in fluorene-containing donor/acceptor polymer system," *J. Mater. Sci. Mater. Electron.*, vol. 30, no. 18, pp. 16892–16902, 2019, doi: 10.1007/s10854-019-01625-1.

[2] J. Rong, T. Zhang, F. Qiu, Y. Zhu, Preparation of Efficient, Stable, and Reusable Laccase-Cu3(PO4)2 Hybrid Microspheres Based on Copper Foil for Decoloration of Congo Red, ACS Sustain. Chem. Eng. 5 (2017) 4468–4477. https://doi.org/10.1021/acssuschemeng.7b00820.

[3] J. Kong, S. Yu, Fourier transform infrared spectroscopic analysis of protein secondary structures, Acta Biochim. Biophys. Sin. (Shanghai). 39 (2007) 549–559. https://doi.org/10.1111/j.1745-7270.2007.00320.x.

[4] I. Lee, H.J. Cheon, M.D. Adhikari, T.D. Tran, K.M. Yeon, M. Il Kim, J. Kim, Glucose oxidase-copper hybrid nanoflowers embedded with magnetic nanoparticles as an effective antibacterial agent, Int. J. Biol. Macromol. 155 (2020) 1520–1531. https://doi.org/10.1016/j.ijbiomac.2019.11.129.

[5] G.A. Parolin, A.S. Menandro, C.G. Barbosa, L.O. Péres, The effect of UV light on luminescent blends, Synth. Met. 253 (2019) 94–99. https://doi.org/10.1016/j.synthmet.2019.05.005.

[6] C.G. de Jesus, R. da Rocha Rodrigues, L. Caseli, L.O. Péres, Conducting polymers modulating the catalytic activity of urease in thin composite films, Colloids Surfaces A Physicochem. Eng. Asp. 654 (2022). https://doi.org/10.1016/j.colsurfa.2022.130136.
